# Tools used to estimate the prevalence of generalized anxiety disorder in populations: a scoping review

**DOI:** 10.1017/S2045796026100432

**Published:** 2026-01-30

**Authors:** Dinara Yessimova, Pauline Sarah Münchenberg, Chisato Ito, Tobias Kurth

**Affiliations:** 1 Department of Health Care Management, Technische Universität Berlin, Berlin, Germany; 2 Institute of Public Health, Charité – Universitätsmedizin Berlin, Berlin, Germany

**Keywords:** diagnostic tools, generalized anxiety disorder, population research, prevalence, scoping review, test accuracy reporting

## Abstract

**Aims:**

Generalized anxiety disorder (GAD) is characterized by persistent worry and physical symptoms, with prevalence estimates ranging from 0.8% to 8%. Researchers utilize various tools, such as standardized diagnostic interviews and self-report questionnaires, to estimate GAD prevalence in population-level studies. However, the diagnostic accuracy of these tools varies greatly. This scoping review aimed to identify the tools used for GAD prevalence estimation and assess the extent to which diagnostic tool accuracy is reported.

**Methods:**

A systematic search was conducted in MEDLINE, Embase and PsycINFO using MeSH terms and keywords related to GAD prevalence. No date restrictions were applied. Studies were eligible if they used nationally or regionally representative samples and defined GAD based on DSM-5, ICD-11 or older case definitions. Studies focusing solely on specific sub-groups were excluded. Data extraction included study characteristics, diagnostic tools and reporting of test accuracy.

**Results:**

A total of 537 studies were initially identified, with 48 meeting inclusion criteria, published between 1994 and 2024. Most studies were conducted in Europe (43.75%) and employed cross-sectional designs (92%). Structured diagnostic interviews were the most commonly used tool (77.08%), although self-report questionnaires gained popularity after 2005. Among the included studies, 62.5% reported test accuracy, often addressing validity and reliability.

**Conclusions:**

Despite the widespread use of diagnostic tools in prevalence studies, test accuracy is not consistently reported, which may impact the reliability of prevalence estimates. The variability in agreement between self-report questionnaires and structured diagnostic interviews highlights the need for transparent reporting of test characteristics to improve the validity of GAD prevalence assessments across populations.

## Introduction

Generalized anxiety disorder (GAD) has a significant impact on affected individuals, causing constant worry about many aspects of life and physical symptoms like restlessness and fatigue (Center for Behavioral Health Statistics and Quality, 2016). GAD typically emerges in late adolescence or early adulthood, yet often with a diagnosis delayed for over a decade due to its subtle onset (Kessler *et al.*, 2001). Even in the absence of comorbid anxiety disorders, GAD results in compromised role functioning, social engagement and overall satisfaction (Mendlowicz and Stein, 2000). Despite its chronicity, periods of symptom remission offer respite, although recurrence is frequent (Yonkers *et al.*, 2003). Early onset and untreated symptoms are linked to heightened disability, further compounded by comorbidities (Bandelow, 2015). Many patients endure years of suffering from GAD before receiving a correct diagnosis, with 45% experiencing symptoms for 2 years or longer before referral to a specialist (Bandelow and Michaelis, 2015).

Before the significant alterations to the GAD definition with the introduction of the Diagnostic and Statistical Manual of Mental Disorders, Third Edition (DSM-III) in 1980, GAD was commonly regarded as a ‘wastebasket’ diagnosis, garnering limited academic and clinical scrutiny (Kendell, 2006; Ruscio *et al.*, 2017). Since the introduction of DSM-III, there have been notable changes in diagnostic criteria, such as reducing symptom criteria items from 18 to 6 in DSM-IV and emphasizing the uncontrollability of worry (Crocq, 2017). In DSM-5, while the definition remains largely similar to its predecessor, one criterion highlights that GAD is typically diagnosed by excluding other anxiety disorders such as panic, phobia, social anxiety or obsessive-compulsive disorder, and it cannot stem directly from stressors or trauma, distinguishing it from adjustment disorders and post-traumatic stress disorder (PTSD; Crocq, 2017).

The diagnostic challenge of GAD is mirrored in global prevalence estimates, as evidenced by Ruscio *et al.* (2017). Conducting a study across 29 nations and regions using the World Health Organization Composite International Diagnostic Interview (CIDI), the authors found a combined lifetime prevalence of GAD of 3.7%, a 12-month prevalence of 1.8% and a 30-day prevalence of 0.8% (Ruscio *et al.*, 2017). The lifetime prevalence estimates varied widely, ranging from less than 1% to approximately 8% of the population being affected (Ruscio *et al.*, 2017).

Diagnosing anxiety disorders, particularly GAD, presents challenges in both clinical settings and population-based studies aimed at estimating disease prevalence. GAD is usually diagnosed in clinical settings through a clinical evaluation by a mental health professional, following the DSM-5 diagnostic criteria (Penninx *et al.*, 2021). In research, especially in large epidemiological population studies, it is more common to use structured or semi-structured interviews and sometimes self-report questionnaires, as these methods are more practical and less costly (Davies *et al.*, 2022; Shabani *et al.*, 2021; Stein *et al.*, 2021). These interviews and tools are considered standard references for diagnosis, but there is no single ‘gold standard’.

Several Standardized Diagnostic Interviews (SDIs) have been developed, with the most prominent ones being the CIDI, the Schedules for Clinical Assessment in Neuropsychiatry (SCAN) and the Mini International Neuropsychiatric Interview (MINI; Verhoeven *et al.*, 2017). Each of these tools reports varying levels of concordance with diagnostic evaluations using Structured Clinical Interview for DSM-5 (SCID-5), ranging from very low to excellent (Verhoeven *et al.*, 2017).

Numerous self-reporting questionnaires, such as the Generalized Anxiety Disorder Scale-7 (GAD-7), Hamilton Anxiety Rating Scale (HAM-A) and Hospital Anxiety and Depression Scale (HADS), among others, are employed to assess anxiety levels, each varying in accuracy (Sapra *et al.*, 2020). Spitzer *et al.* (2006) introduced the GAD-7 as a concise instrument aimed at identifying potential instances of GAD. However, it is crucial to note that these screening tools alone are not intended and not sufficient for diagnosing anxiety disorders, necessitating a confirmatory diagnostic evaluation if a screening test indicates the presence of such a disorder (O’Connor *et al.*, 2023).

All SDIs and self-report questionnaires used to assess the prevalence of GAD exhibit varying degrees of agreement with clinical diagnoses. For example, Verhoeven *et al.* (2017) found that the MINI questionnaire reported a 1.61 times higher prevalence of anxiety disorder compared to clinical assessments, suggesting a notable number of false positive cases in real-life datasets (*n* = 7,016). Additionally, the widely used cut-off score of 10 for the GAD-7 questionnaire yields a sensitivity of 0.74 (with a range of 0.61–0.84) and a specificity of 0.83 (with a range of 0.68–0.92). It indicates that while the GAD-7 is effective at identifying individuals with GAD, it may also identify some individuals who do not have GAD as positive, highlighting the importance of further investigation and clinical follow-up to ensure accurate diagnoses (Plummer *et al.*, 2016). The varying degrees of accuracy of the tools highlight the necessity for careful scrutiny. Given the disparities between tool-based assessments and clinical diagnoses, it is crucial to approach GAD prevalence data with caution and discernment.

Despite the importance of accurately estimating the prevalence of GAD at regional or national levels, there is a lack of comprehensive research that systematically evaluates the array of tools employed for this purpose. Such an evaluation would provide an overview of the existing methods. The extent to which existing prevalence studies use SDIs or self-report tools is unclear, revealing a critical gap in research. To address this gap, more research is needed to understand the methods used in population-level prevalence studies and how they affect accuracy and reliability.

Hence, this study aims to investigate tools used for estimating the prevalence of GAD, defined by DSM-5, ICD-11 or older case definitions, at a population level, specifically examining the choice between self-reported questionnaire-based screening tools and SDIs. By providing an overview of the tools commonly used in epidemiological studies for prevalence estimation, we seek to understand the prevailing trends in research choices. Additionally, our goal is to assess whether researchers consider and report the test accuracy of their chosen tool in their publications. The findings can also inform guidelines for standardizing methodology and reporting practices for prevalence estimation studies.

## Methods

We conducted a scoping review to capture the extent to which researchers use SDIs and self-report questionnaires in epidemiological studies that estimate the prevalence of GAD. This scoping review’s search and reporting followed the guidelines set forth by the Preferred Reporting Items for Systematic reviews and Meta-Analysis extension for Scoping Reviews (PRISMA-ScR; Tricco *et al.*, 2018) and the JBI methodology for scoping reviews (Peters *et al.*, 2020).

The protocol for this study was registered on the Open Science Framework and is readily accessible at https://doi.org/10.17605/OSF.IO/QUHJE. PRISMA-ScR checklist is provided in Supplementary material 2.

### Search strategy

A literature search was performed in MEDLINE, Embase and PsycINFO. Employing Medical Subject Headings (MeSH terms) and keywords associated with ‘Generalized anxiety disorder’, ‘GAD’, ‘Prevalence’ and ‘General population’, the search strategies were defined and translated to each of the selected databases. No specific date range was enforced in the search to capture differences in the tools used to estimate the prevalence of GAD at the population level. In addition, reference lists of included studies were evaluated for potential studies that could also be included in the review. Full search strategies can be found in Supplementary material 1. The initial search was conducted on 8 November 2023, followed by an additional search on 23 February 2025, to identify any newly published articles.

### Study/source of evidence selection

The eligibility criteria of the scoping review required studies to utilize nationally or regionally representative samples for population-level estimation of GAD prevalence, and the GAD to be defined based on DSM-5, ICD-11 or older case definitions. Detailed inclusion criteria can be found in the registered protocol. The review examined all population/national-level studies but excluded studies focusing solely on specific sub-groups of the population. Systematic reviews, meta-analyses, case reports and intervention studies were not included in the review. Two reviewers (D.Y. and P.S.M.) assessed all studies individually using Rayyan software (Ouzzani *et al.*, 2016) and subsequently reached a consensus on whether to include each one.

### Data extraction and analysis plan

Two reviewers (D.Y. and P.S.M.) independently extracted data and collectively reached a consensus on the extracted information. The review synthesized evidence by capturing specific information, which was organized into a table format. The following information was captured: (i) authors, (ii) year of publication, (iii) country, (iv) study design, (v) diagnostic tool(s) utilized and (vi) reporting of accuracy of diagnostic tool(s).

## Results

**Figure 1.**
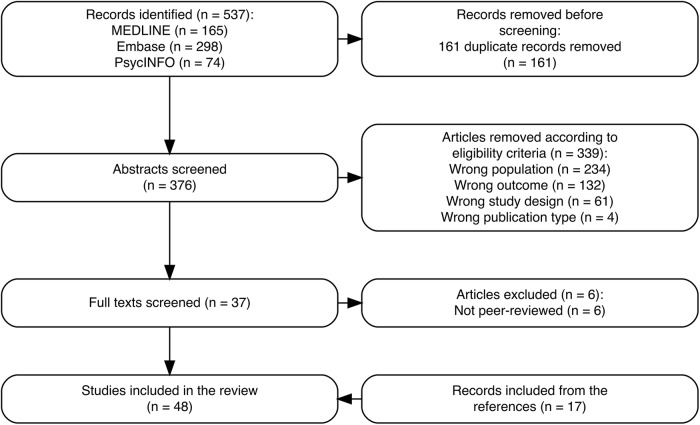
PRISMA flow diagram: diagnostic tools in GAD prevalence estimation.

The search strategies initially yielded 537 studies, from which 161 duplicates were identified and, therefore, removed. Following a thorough screening of 376 abstracts, 37 studies were deemed eligible for full-text screening based on the predefined criteria. A total of 339 studies were excluded for not meeting the inclusion criteria, with detailed reasons recorded by reviewers in the Rayyan screening tool. Some studies were excluded for multiple reasons. Six articles were also excluded because they were not published in peer-reviewed journals. Seventeen studies were identified through a reference list screening of the articles that were included, resulting in a total of 48 articles included in the review. Figure 1 depicts the process of study identification, exclusion and inclusion in the final review in the format of the PRISMA flow diagram (Tricco *et al.*, 2018). Table 1 provides an overview of the included studies.

**Table 1.** Characteristics of studies included in the review

Authors	Country	Study design	Study cohort	Utilized tool	Test accuracy reporting	Details of reporting
Bijl *et al.* (1998)	Netherlands	Cross-sectional	7,076	WHO – Composite International Diagnostic Interview (WHO-CIDI)	Not mentioned	
Bunting *et al.* (2013)	UK	Cross-sectional	4,340	WHO-CIDI	Not mentioned	
Burstein *et al.* (2014)	USA	Cross-sectional	10,123	WHO-CIDI	Not mentioned	
Caballero *et al.* (2009)	Spain	Cross-sectional	20,347	Clinical diagnosis	Not mentioned	
Carter *et al.* (2001)	Germany	Cross-sectional	4,181	Stage 1 – Composite International Diagnostic-Screener (CID-S), Stage 2 – Munich CIDI	Yes, in Methods	Referenced a specific validation study. **Stage 1:** Test–retest reliability showing kappa values of 0.64–0.92 and an overall sensitivity of 85.3% for the included disorders. **Stage 2:** Test–retest reliability showing kappa values of 0.56–0.81 for main diagnostic categories, and procedural validity compared to clinical consensus diagnoses yielding kappa values ranging 0.50–0.96, excluding psychotic disorders.
Chang *et al.* (2019)	Singapore	Cross-sectional	6,126	WHO-CIDI	Not mentioned	
Cho *et al.* (2015)	South Korea	Cross-sectional	6,022	Korean CIDI	Yes, in Methods	Referenced a specific validation study.
Chong *et al.* (2012)	Singapore	Cross-sectional	6,616	WHO-CIDI	Yes, in Methods	Referenced a specific validation study. Demonstrates high accuracy in diagnosing mental disorders, validated against the SCID, with moderate to good concordance found for lifetime prevalence estimates of most disorders across multiple countries.
Clark *et al.* (2007)	UK	Longitudinal	9,377	Revised Clinical Interview Schedule (CIS-R), teacher-rated Bristol Social Adjustment Guides	Yes, in Methods	Referenced a specific validation study. **Stage 2:** Internalizing and externalizing problems at ages 7 and 11 years were evaluated using the teacher-rated Bristol Social Adjustment Guides, revealing reliable scales with Cronbach’s *α* coefficients ranging from 0.69 to 0.73.
Comer *et al.* (2011)	USA	Cross-sectional	43,000	Alcohol Use Disorder and Associated Disabilities Interview Schedule – DSM-IV Version (AUDADIS-IV)	Yes, in Methods	Referenced a specific validation study. Reported to demonstrate good retest reliability, comparable to or exceeding other diagnostic interviews, and is designed for lay interviewers, ensuring robust measurement of substance use and mental disorders in large-scale surveys.
Erhardt *et al.* (2023)	Germany	Cross-sectional	93,002	Generalized Anxiety Disorder Scale-7 (GAD-7)	Yes, in Methods	Referenced a specific validation study. The GAD-7 has high sensitivity (89%) and specificity (82%) for diagnosing GAD with a cut-off score of ≥10 points, while using a cut-off of ≥8 points yields acceptable diagnostic accuracy in the general population (sensitivity: 83%, specificity: 84%).
de Graaf *et al.* (2005)	Netherlands	Cross-sectional	7,076	WHO-CIDI	Yes, in Methods	Referenced a specific validation study. Reported that CIDI demonstrates acceptable reliability and validity across various diagnoses, allowing for the assessment of one-month, 12-month and lifetime prevalence rates.
Grant *et al.* (2005)	USA	Cross-sectional	43,093	AUDADIS-IV	Yes, in Methods	Referenced a specific validation study. Reported that reliability of repeated testing was acceptable for GAD (kappa = 0.42).
Hajebi *et al.* (2018)	Iran	Cross-sectional	7,886	WHO-CIDI	Not mentioned	
Hunt *et al.* (2004)	Australia	Cross-sectional	10,641	WHO-CIDI	Yes, in Methods	Referenced a specific validation study. Reported that while specific reliability data for Version 2.1 are not available, earlier versions of CIDI have shown reliability.
Hyland *et al.* (2020)	Ireland	Cross-sectional	1,041	GAD-7	Yes, in Methods and Discussion	Referenced a specific validation study. Using a score of ≥10, the survey demonstrated 89% sensitivity and 82% specificity, potentially overestimating anxiety and depression prevalence due to its self-report nature compared to clinical interviews.
Ishikawa *et al.* (2018)	Japan	Cross-sectional	2,450	WHO-CIDI	Yes, in Methods	Referenced a specific validation study. Simply reported that the accuracy and trustworthiness of the WHO-CIDI 3.0 and its Japanese version are discussed in other sources.
Jacobi *et al.* (2004)	Germany	Cross-sectional	7,124	Stage 1 – Composite International Diagnostic-Screener (CID- S), Stage 2 – Munich CIDI	Not mentioned	
Jayasankar *et al.* (2023)	India	Cross-sectional	34,802	Mini International Neuropsychiatric Interview (MINI)	Not mentioned	
Judd *et al.* (1998)	USA	Cross-sectional	8,098	WHO-CIDI	Not mentioned	
Kadri *et al.* (2007)	Morocco	Cross-sectional	800	MINI	Yes, in Methods	Referenced a specific validation study. Reported that study utilized a validated Moroccan Arabic version of the MINI to assess mental health conditions according to DSM IV criteria.
Kessler *et al.* (2005)	USA	Cross-sectional	5,692	WHO-CIDI	Not mentioned	
Khan *et al.* (2018)	India	Cross-sectional	1,700	MINI	Not mentioned	
Kujanpää *et al.* (2016)	Finland	Cross-sectional	5,480	GAD-7	Yes, in Methods	Referenced a specific validation study. Reported that the GAD-7, with a cut-off score of 10 or higher, demonstrates high validity, with a sensitivity of 89% and a specificity of 82%. While the Finnish translation achieved 100% sensitivity and 83% specificity with a cut-off score of 7 points among healthcare high utilizers, the recommended cut-off of 10 points showed better specificity (95%) but lower sensitivity (67%) in a larger international study.
Lee *et al.* (2007)	Hong Kong	Cross-sectional	3,034	Self-reported questionnaire with three questions	Yes, in Discussion	Referenced a specific validation study. Reported that there was no study conducted on the tool’s reliability and validity.
Leray *et al.* (2011)	France	Cross-sectional	36,105	MINI	Not mentioned	
Levecque (2006)	Belgium	Cross-sectional	9,413	Symptom Checklist – 90	Yes	Did not reference a specific validation study. Reported that since there is no validity for SCL-90, the authors decided to use a 90% threshold.
Matsuyama *et al.* (2024)	Japan	Cross-sectional	20,009	GAD-7	Yes, in Methods	Referenced a specific validation study. Report that GAD-7, with a cutoff of 10, showed strong psychometric properties globally (89% sensitivity and 82% specificity). Its Japanese version demonstrated similar reliability (88% sensitivity and 82% specificity).
McDowell *et al.* (2018)	Ireland	Longitudinal	3,236	Wave 1 – Penn State Worry Questionnaire (PSWQ-A), Wave 2 – CIDI Short Form (CIDI-SF)	Yes, in Methods	Referenced a specific validation study. **Stage 1**: Report that the PSWQ-A showed strong reliability (Cronbach’s *α* = 0.94). A score of 23 or higher indicates probable GAD, with 75.7% sensitivity and 92.5% specificity. **Stage 2**: CIDI-SF has been shown to correctly classify CIDI cases of GAD, with an accuracy of 99.6%.
McEvoy *et al.* (2011)	Australia	Cross-sectional	8,841	WMH-CIDI (3.0, Computer Assisted Personal Interview version 20)	Yes, in Discussion	Did not reference a specific validation study, but reported that WMH-CIDI may not be sensitive to cultural differences in mental disorder phenotypes.
Mohammadi *et al.* (2005)	Iran	Cross-sectional	25,180	Social Avoidance and Distress Scale (SADS)	Yes, in Methods	Referenced a specific validation study. Report a strong agreement between interviewers. For test–retest interviewers, the Cohen kappa was 0.87.
Murcia *et al.* (2015)	France	Cross-sectional	13,648	MINI	Not mentioned	
Pignon *et al.* (2018)	France	Cross-sectional	38,694	MINI	Yes, in Methods	Referenced a specific validation study. Report that it has been validated in the general population and has good validity, reliability (inter-rater and test–retest), sensitivity and specificity.
Remes *et al.* (2017)	UK	Cross-sectional	18,494	Health and Life Experiences Questionnaire (HLEQ)	Not mentioned	
Ruscio *et al.* (2017)	26 countries	Cross-sectional	147,261	WHO-CIDI	Yes, in Discussion	Did not reference a specific validation study, but stated that clinical reappraisal studies have shown good concordance with the SCID, but indicated that CIDI-based prevalence estimates tend to be conservative.
Schoevers *et al.* (2003)	Netherlands	Longitudinal	4,051	MINI, Geriatric Mental Examination (GMS-AGECAT)	Not mentioned	
Shen *et al.* (2006)	China	Cross-sectional	5,201	Chinese CIDI	Yes, in Methods	Checked the validity of the tool themselves, but did not provide any specifics. Report that an expert panel evaluated content validity, tested the instrument with Chinese patients and revised it for laypeople’s comprehension.
Shevlin *et al.* (2020)	UK	Cross-sectional	2,025	GAD-7	Yes, in Methods	Referenced a specific validation study. Report that a cut-off score of 10 was used, with a reported sensitivity of 89% and a specificity of 82%.
Skapinakis *et al.* (2013)	Greece	Cross-sectional	4,894	CIS-R	Yes, in Methods	Referenced a specific validation study. Reported that a CIS-R score of ≥12 is typically the cut-off for ‘clinically significant’ psychiatric morbidity.
Stein and Heimberg (2004)	Canada	Cross-sectional	8,116	University of Michigan CIDI	Yes, in Methods	Referenced a specific validation study. Reported that the field trials of the CIDI show it to be a highly reliable instrument for almost all diagnoses except mania and psychosis.
Subramaniam *et al.* (2019)	Singapore	Cross-sectional	6,126	WHO-CIDI	Not mentioned	
Taillieu *et al.* (2018)	Canada	Cross-sectional	15,981	WHO-CIDI	Yes, in Discussion	Did not reference a specific validation study. Reported that there are several limitations of the CIDI in detecting GAD in specific populations.
van Loo Hm *et al.* (2021)	Netherlands	Cross-sectional	146,315	MINI	Not mentioned	
Vollebergh *et al.* (2001)	Netherlands	Longitudinal	7,076	WHO-CIDI	Yes, in Methods	Referenced a specific validation study. Reported that CIDI is globally utilized, with WHO research showing high inter-rater and test–retest reliability, along with acceptable validity across most diagnoses.
Wells *et al.* (2006)	New Zealand	Cross-sectional	12,992	WHO-CIDI	Not mentioned	
Wittchen *et al.* (1994)	USA	Cross-sectional	8,098	WHO-CIDI	Yes, in Methods	Referenced a specific validation study. Reported that reliability and validity of the GAD section in the CIDI have been extensively studied in international research.
Xia *et al.* (2023)	China	Cross-sectional	10,824	GAD-7	Yes, in Methods and Discussion	Referenced a specific validation study. Reported that GAD-7 has been widely employed in China and has shown good retest reliability and validity. However, the authors acknowledge that self-report scales may not offer the same precision as clinical diagnosis.
Yu *et al.* (2018)	China	Cross-sectional	36,806	GAD-7	Yes, in Methods	Referenced a specific validation study. Reported that GAD-7 has good reliability, high sensitivity (89%) and specificity (82%).

The article publication year ranged from 1994 to 2024. Only one study (Ruscio *et al.*, 2017) was a cross-national study conducted across 26 countries. Most studies were carried out in Europe (21/48, 43.75%). Most studies (44/48, 91.6%) were cross-sectional, while four were longitudinal. On average, studies had around 19,552 participants, ranging from 800 to 147,261.

### Utilized tools

**Figure 2.**
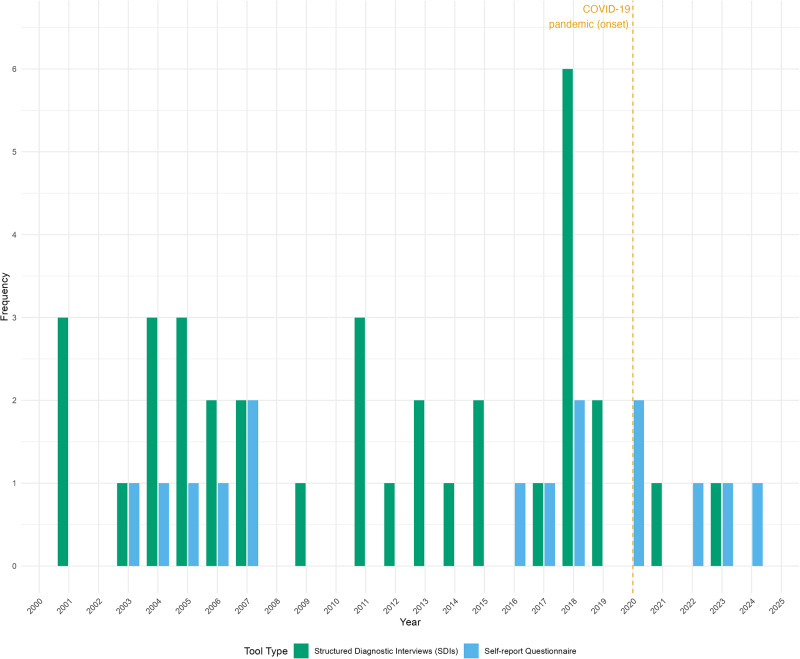
Use of tool types for GAD estimation over time.

Table 2 illustrates the distribution of tools used in the included studies, while Figure 2 further categorizes these tools by year of publication. The majority of studies (37/48, 77.08%) employed diagnostic interviews for assessing GAD prevalence, with CIDI being the most frequently used tool (25/48, 52.08%), followed by MINI (7/48, 14.58%) and other clinical interviews (4/48, 8.33%). Additionally, 15 studies (16/48, 33.33%) used only self-report questionnaires to estimate prevalence or as a first step in longitudinal studies. The GAD-7 was the most commonly used self-reporting tool (7/48, 14.58%). In three longitudinal studies, self-report questionnaires were utilized in at least one stage of the study. Before 2005 (1994–2004), all studies used diagnostic interviews; however, after 2005, the popularity of self-reported tools grew rapidly. Since 2020, four of the seven population-based studies included in this review have used the GAD-7 to assess the prevalence of GAD, as shown in Figure 2.

**Table 2.** Type of the tool used in the studies

Tool	*N*	%^a^
Diagnostic interviews	37	77.08
WHO – Composite International Diagnostic Interview (CIDI)	25	52.08
Mini International Neuropsychiatric Interview (MINI)	7	14.58
Other versions of clinical interviews^b^	4	8.33
Self-reported questionnaires	16	33.33
Generalized Anxiety Disorder Scale-7 (GAD-7)	7	14.58
Other self-reported questionnaire tools^c^	9	18.75

a The percentages presented in the table were calculated using the total number of articles (48) as the denominator.

b CIS-R and AUDADIS-IV.

c CID-S, Bristol Social Adjustment Guides, self-reported questionnaire with three questions, PSWQ-A, SADS, HLEQ and GMS-AGECAT.

### Test accuracy reporting

Out of 48 articles included in the review, 30 (62.5%) studies commented or reported on test accuracy in methods, discussion or both sections, but the remaining 18 (37.5%) studies did not. These studies typically mentioned the validity and reliability of the tools, often using measures like the kappa statistic, or they provided references to respective validation studies. In non-English speaking countries, 17 (35.42%) studies also addressed the translation and adaptation of assessment tools to local languages to varying extents. Additionally, four studies acknowledged the imperfect accuracy of the tools in their discussion sections, noting potential limitations such as reduced accuracy of prevalence estimates in certain populations.

All six studies employing GAD-7 as their screening tool for prevalence estimation reported test accuracy in the methods section. Additionally, five out of six studies that used GAD-7 provided explanations for the chosen cut-off score and the sensitivity and specificity of the tool at that cut-off.

## Discussion

This scoping review aimed to summarize the methods used in population-level GAD prevalence studies and assess how often researchers reported the accuracy of the tools they used. Among the 48 included studies, a clear methodological trend was observed: while SDIs were commonly used for prevalence estimation, there has been a noticeable shift in recent years towards the use of self-report questionnaires or shorter diagnostic formats.

One of the driving factors behind the popularity of self-report tools, such as the GAD-7 and GAD-2, is their practicality. These tools are quick, low cost and easy to administer, making them attractive for large-scale studies and especially for use in primary care, where time constraints are common (Salinas *et al.*, 2022). Our findings reflect this shift, showing increased use of self-report questionnaires in more recent studies. However, it is important to emphasize that a positive screen on a self-report tool does not equate to a clinical diagnosis. These tools are designed for screening purposes and should ideally be followed by a structured clinical interview for confirmation. Without this, prevalence estimates based solely on self-reported symptoms risk being either inflated or underestimated.

Test accuracy, particularly sensitivity and specificity, remains a crucial yet inconsistently reported aspect. Although more studies are beginning to mention these metrics, especially when using self-report tools, very few incorporate them analytically. For example, the GAD-7 has a sensitivity of 0.74 at the conventional cut-off of 10 (Plummer *et al.*, 2016), suggesting a significant proportion of true cases may go undetected. Moreover, these tools may also capture symptoms of other mental health conditions, such as depression, PTSD or panic disorders (Craske *et al.*, 2017; O’Connor *et al.*, 2023), further muddying the accuracy of prevalence estimates. Therefore, studies must more clearly report and account for the diagnostic limitations of the tools they use.

The COVID-19 pandemic accelerated the use of self-report questionnaires, likely due to the need for remote data collection and limited in-person interaction. Of the five studies published after 2020, four used the GAD-7 and only one used a structured interview (MINI). While this shift was perhaps necessary in a crisis context, it underscores the ongoing reliance on tools whose diagnostic precision is limited. Notably, none of these recent studies adjusted their analyses for test inaccuracies, despite acknowledging the limitations of their tools.

A recent article by Kohrt *et al.* (2025) highlights the global challenges of accurately diagnosing mental health conditions, particularly in low- and middle-income countries. They argue that relying heavily on self-report tools, which tend to produce high false-positive rates, can lead to inflated and misleading prevalence figures. Kohrt *et al.* (2025) recommend combining statistical adjustment of self-report data with structured clinical interviews and adopting contextually relevant ‘good-enough’ diagnostic categories. This approach aligns with our findings. While many of the studies in our review continue to use SDIs, we also observed a growing reliance on self-report questionnaires, often without addressing their diagnostic limitations. These limitations should not only be acknowledged but also incorporated into the analysis, reinforcing the need for more rigorous and methodologically sound approaches in prevalence research.

This need is further supported by methodological work of Fischer *et al.* (2023), which demonstrates the value of incorporating test accuracy into prevalence estimation using Bayesian latent class models. Applying such methods could help generate more valid and nuanced estimates of mental disorder prevalence. We therefore advocate for both reporting test accuracy and incorporating it into analytical frameworks. Strengthening this aspect of methodology would improve the reliability of findings and provide more actionable evidence for mental health policy and practice.

### Limitations

Our scoping review has some limitations. Firstly, despite efforts to create a comprehensive search strategy, it is possible that some relevant studies may have been missed, especially if they were published in non-indexed journals or were not available in the selected databases. Additionally, restricting the search to specific databases may have omitted relevant studies indexed elsewhere. Even though there was no restriction on our search strategy in terms of the geographical region or language used in the publication, most of the studies included in the review were conducted in middle- to high-income countries, which can also affect the choice of the tool used to capture the prevalence of GAD.

## Conclusion

This scoping review provides a comprehensive overview of the tools utilized in assessing the prevalence of GAD at a population level. While SDIs are still widely utilized, there is a noticeable trend towards using self-report questionnaires, potentially attributable to their convenience and cost-effectiveness. However, despite the growing number of prevalence studies, few assess or incorporate the test accuracy of the tools into their analyses.

It is imperative to acknowledge that SDIs and self-report questionnaires may yield inaccurate GAD prevalence estimates compared to clinical diagnosis settings and that the agreement between the two types of diagnostic tools varies across different populations. This review underscores the necessity for transparent reporting of test accuracies to enhance the reliability and validity of prevalence estimates. Moreover, where possible, researchers should consider applying methods to incorporate test accuracy into prevalence estimation, particularly when self-report tools are used. Future research should prioritize standardized reporting of test characteristics to improve comparability across studies and inform more accurate public health strategies for GAD prevention and intervention.

## Supporting information

10.1017/S2045796026100432.sm001 Yessimova et al. supplementary material Yessimova et al. supplementary material

## Data Availability

All extracted data are presented in the article, and the full search strategy can be accessed in Supplementary material 1.

## Supplementary material

The supplementary material for this article can be found at https://doi.org/10.1017/S2045796026100432.
